# Natural killer cells: a future star for immunotherapy of head and neck squamous cell carcinoma

**DOI:** 10.3389/fimmu.2024.1442673

**Published:** 2024-08-21

**Authors:** Shuyan Dong, Ming Zhao, Jin Zhu, Ting Li, Mingze Yan, Kaixun Xing, Peng Liu, Shan Yu, Jian Ma, Hongjiang He

**Affiliations:** ^1^ Department of Head and Neck Surgery, Harbin Medical University Cancer Hospital, Harbin, China; ^2^ Department of Gastroenterology, Clinical Medical College and the First Affiliated Hospital of Chengdu Medical College, Chengdu, China; ^3^ Department of Pathology, Xi’an Daxing Hospital, Xi’an, China; ^4^ Laboratory of Medical Genetics, Harbin Medical University, Harbin, China; ^5^ Department of Pathology, Second Affiliated Hospital of Harbin Medical University, Harbin, China; ^6^ Department of General Surgery, Second Affiliated Hospital of Harbin Medical University, Harbin, China; ^7^ Department of Immunology, Harbin Medical University, Harbin, China

**Keywords:** NK cell, interleukin, HNSCC, immunotherapy, cetuximab

## Abstract

The interplay between immune components and the epithelium plays a crucial role in the development and progression of head and neck squamous cell carcinoma (HNSCC). Natural killer (NK) cells, one of the main tumor-killing immune cell populations, have received increasing attention in HNSCC immunotherapy. In this review, we explore the mechanism underlying the interplay between NK cells and HNSCC. A series of immune evasion strategies utilized by cancer cells restrict HNSCC infiltration of NK cells. Overcoming these limitations can fully exploit the antineoplastic potential of NK cells. We also investigated the tumor-killing efficacy of NK cell-based immunotherapies, immunotherapeutic strategies, and new results from clinical trials. Notably, cetuximab, the most essential component of NK cell-based immunotherapy, inhibits the epidermal growth factor receptor (EGFR) signaling pathway and activates the immune system in conjunction with NK cells, inducing innate effector functions and improving patient prognosis. In addition, we compiled information on other areas for the improvement of patient prognosis using anti-EGFR receptor-based monoclonal antibody drugs and the underlying mechanisms and prognoses of new immunotherapeutic strategies for the treatment of HNSCC.

## 1 Introduction

Head and neck squamous cell carcinoma (HNSCC) is the sixth most common malignancy worldwide, with 379,000 deaths annually (1, 2). HNSCC originates in the epithelium of the oral cavity, hypopharynx, oropharynx, and larynx (3) and tends to escape the immune system, with a poor prognosis (4). The use of immune checkpoint inhibitors has resulted in significant outcomes for some HNSCC patients. Although these drugs fail in most cases, there is still interest in targeting immune effector cells such as natural killer (NK) cells for immunotherapy. NK cells play an important role in tumor immune surveillance. Without prior sensitization, NK cells can quickly recognize and attack cancer cells (5). However, in some cases, intratumoral NK cells are dysfunctional and contribute to tumor growth. In contrast, when infiltrating activated NK cells increase in tumors, especially HNSCC, the clinical prognosis is significantly better (6). Therefore, NK cells play a crucial role in tumor management, prognosis, growth, and metastasis (7). In the last few years, NK cell immunotherapy has provided a different view of head and neck tumors. Cetuximab has received increasing attention because it can activate NK cells and produce durable responses. In this review, we introduce HNSCC-based NK cell immunotherapy from the perspective of modifying NK cell activation, proliferation, cell engineering, and inhibition of immune escape in HNSCC.

## 2 A brief introduction of NK cells

NK cells originating from CD34^+^ hematopoietic stem cells are the main inherent mediated antitumor and antiviral response lymphocyte subsets, and under a stimulus, they produce interferon γ (IFN-γ) and tumor necrosis factor α (TNF-α) (8, 9). NK cells express a variety of surface markers, such as CD56. According to the CD56 expression level, they could be divided into CD56^dim^ and CD56^bright^ subsets, which are mainly present in peripheral blood and tissues (10). CD16 is an NK cell surface marker. CD16 enables NK cells to exhibit antibody-dependent cell cytotoxicity (ADCC), as demonstrated by the minimal ADCC effect of CD56^bright^CD16^−^ NK cells (11, 12). In contrast, CD56^dim^ CD16^+^ NK cells can be mediated by infected cells and/or the continuous destruction of malignant cells, mainly through immune synapses that shed pre-assembled cellular lyses containing granzyme B and perforin, ultimately inducing target cell apoptosis (13).

Multiple receptors have been found to transmit signals that inhibit or activate NK cells. NKG2D is an agonist receptor on the NK cell surface, and its activation can be initiated by various NKG2D ligands (**Table 1**) (23). The immunotherapeutic approach of the NKG2D–NKG2DL axis is an appealing therapeutic target for cancer immunotherapy (24, 25). When cells are in a pathological state, surface HLA expression is usually downregulated to evade recognition by T cells. Target cells lacking HLA ligand expression require NK cells to inhibit the receptor and express ligands that activate the receptor, which may lead to NK cell activation without inhibitory signals (26). However, the role of NK cells in HNSCC remains unclear. This may be related to HNSCC cells producing various cytokines or modulating the signal transduction of different receptors, which is discussed in detail later in this review.

**Table 1:** NK cell receptors and ligands.

	NK receptor	Ligands	Ref
Activating	NKG2C/CD94	HLA-E	(14)
	NKp30	BAT-3/BAG6	(15, 16)
	NKp44	PDGF-DD	(17)
	CD16	FcR	
Co-stimulatory	NKG2D	MICA/B, ULBPs	
	NKp46	Viral HA, HS, GAGs	(18–20)
	NKp80	AICL1	
Inhibitory	NKG2A/CD94	HLA-E	(21)
	KIR2DL1	HLA-C, group 2	(22)
	KIR2DL2/3	HLA-C, group 1	(22)
	KIR3DL1	HLA-Bw4	(22)
	KIR3DL2	HLA-A3, A11	(22)
	CD96		

NK, natural killer.

## 3 NK cell recognition and killing HNSCC

**Figure 1:**
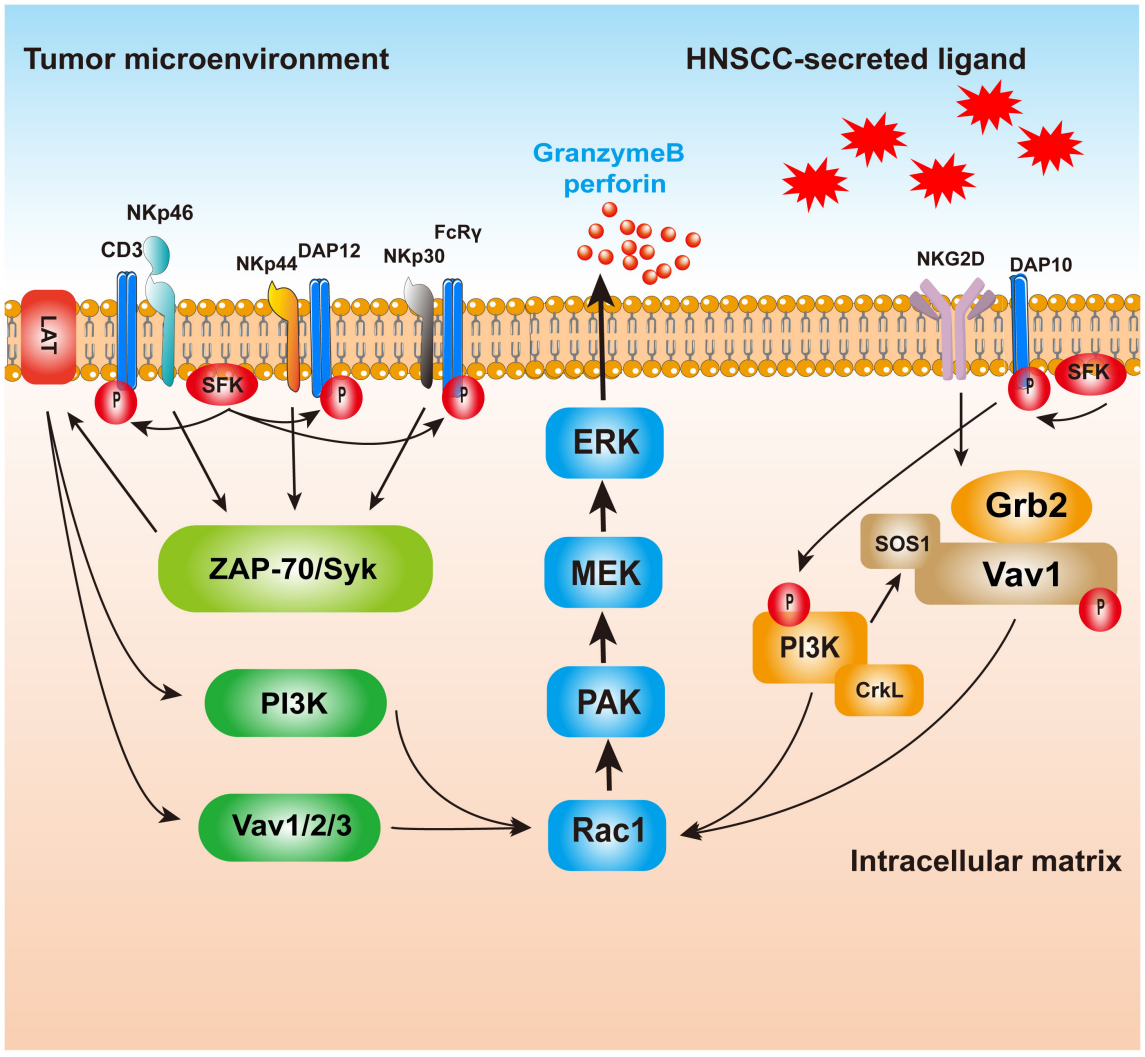
The killing mechanism of NK cells on HNSCC. Ligand binds to receptors on NK cells, and the NCR will raise and activate the downstream molecules with the help of the LAT; PAK1 MEK–Erk signaling pathway activation of MAPK signaling pathways induced enzyme release particles, HNSCC. NKp44, however, may be associated with inhibitory signaling, but the mechanism is still unclear. NKG2D activated, combined with PI3K and Grb2–Vav1–SOS1, raised the Ras family, and finally passed PAK1 MEK–Erk signaling pathway activation of MAPK signaling pathways in order to realize cell toxicity. NK, natural killer; HNSCC, head and neck squamous cell carcinoma; NCR, natural cytotoxicity receptor.

Major histocompatibility complex class I molecule (MHC I), also called human leukocyte antigen (HLA), is expressed on the surface of healthy cells and interacts with killer cell immunoglobulin-like receptor (KIR) on NK cell surfaces, inhibiting them through KIR–KIRL interactions (27). The KIR receptor binds to the ligand, and the intracellular ITIM sequence phosphorylates and enrolls SHP-1/SHP-2, which downregulates the phosphorylation of signaling molecules downstream of the activated receptor, thereby inhibiting NK cell function (28). Decreased MHC I expression on the surface of HNSCC cells leads to NK cell activation (29). NK cells are also auto-activated via the interplay between CD16 and the Fc structural domain of IgG antibodies and release cytokines and cytotoxic particles when interacting with tumor cells through the Fab structural domain of the antibodies (30). Furthermore, natural cytotoxicity receptor (NCR) is also a major activated receptor for NK cells, including NKp46, NKp44, and NKp30 (**Figure 1**) (31). NKp46 transmits activation signals via ITAM-associated receptors. Upon ligand binding, NKp46 binds to the junctional proteins CD3ze and FcϵRIγ, and the junctional ITAMs are phosphorylated and activated by SH2 recruitment. Tyrosine kinases activate downstream factors (such as PLC, PI3K, Vav1, Vav2, and Vav3) (32, 33). PI3K and Vav1 recruit small G proteins, and the PAK1 MEK–ERK signaling pathway induces a phosphorylation cascade that activates NK cells to kill HNSCC cells (28). Some HNSCC cells routinely overexpress heat shock protein 70 (Hsp70) and deliver it to the cell surface for recognition by pre-activated NK cells (34). These different effects of NK cells on tumor cells expressing Hsp70 or not expressing Hsp70 on the envelope were compared and analyzed. The results showed that tumor cells expressing Hsp70 were more sensitive to NK-mediated lysis (35). NK cells kill HNSCC cells expressing Hsp70 by releasing granzyme B. In 2014, Gehrmann et al. reported that Hsp70 may activate NK cells by inducing NKG2D expression (36).

## 4 Immune escape of NK cells

**Figure 2:**
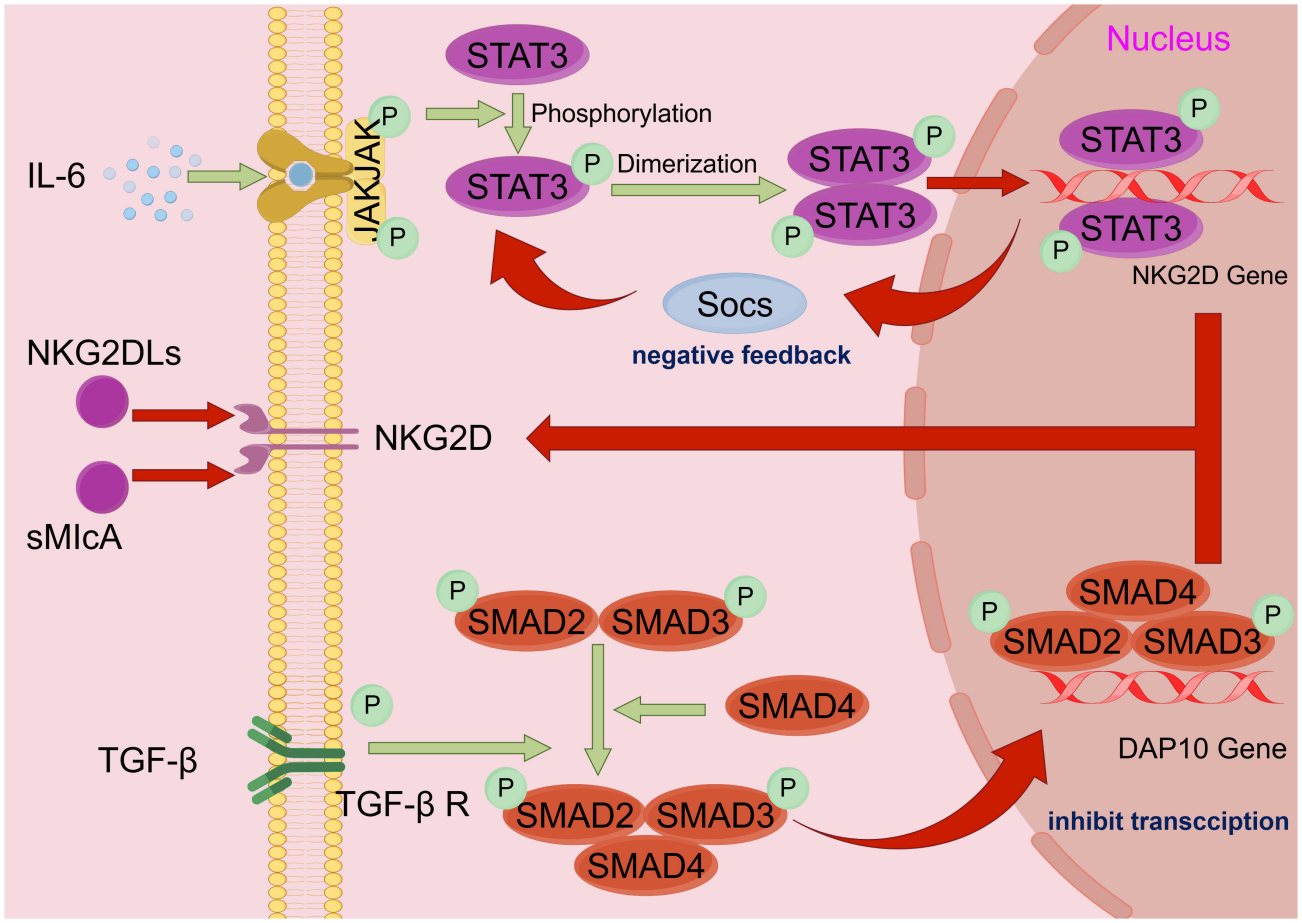
HNSCC immune escape mechanism. Schematic diagram of the mechanism by which HNSCC escapes NK cell immune surveillance: tumor cell escape mostly acts by affecting NKG2D, the surface receptor of NK cells. i) IL-6 activates the JAK/STAT3 pathway, causing STAT3 to phosphorylate and polymerize into a dimer into the nucleus, thereby inhibiting gene expression of the NKG2D receptor. ii) HNSCC secretes inhibitory ligands of NKG2D (e.g., NKG2DLs and sMIcA) to inactivate NKG2D. iii) TGF-β binds to TGF-βR on the NK cells surface to activate downstream SMAD2, SMAD3, and SMAD4 polymerization and then enters the nucleus to inhibit DAP 10 gene transcription, indirectly reducing the quantity of NKG2D on the cell surface. By Figdraw. HNSCC, head and neck squamous cell carcinoma; NK, natural killer.

Various HNSCC-derived cytokines have been reported to inhibit NK cell function by inhibiting agonist receptor signaling or enhancing inhibitory receptor signaling (**Figure 2**) (37). HNSCC-derived IL-6 is one of the most crucial cytokines that mediate NK cell inactivation (38). IL-6 activates JAK/STAT3 signaling in NK cells to reduce NKG2D transcription, thereby inhibiting NK cells (39–41). TGF-β is also a well-known immunosuppressive factor, and in a 2004 trial in Oregon, the level of TGF-β was also higher in HNSCC tumors from 32 patients with sleep apnea than in five normal oropharyngeal tissues (42). TGF-β not only stimulates angiogenesis to promote HNSCC growth but immunizes HNSCC from NK cell immunosurveillance by inhibiting NKG2D (43). Increased binding of plasma-soluble major histocompatibility complex class I chain-associated peptide A (sMICA) to NKG2D inhibits its function in some patients with HNSCC. TGF-β1 potentiates negative regulatory sMICA effect on NKG2D expression in NK cells. In freshly purified patient and healthy NK cell control experiments, the human HNSCC cell line SCC-4 (ATCC: CRL-1624) showed synergistic inhibition of NKG2D expression under the action of multiple factors, especially TGF-β1 (44).

Inhibition of NK cell surface agonist receptors enables immune escape of HNSCC cells. NKG2D is a C-type lectin-like activation receptor expressed on NK cells as well as TCRγδ^+^ and CD8^+^ TCRαβ^+^ T cells that stimulate naïve T-cell activation and provokes cytotoxicity without T-cell receptor (TCR) attachment (45). NKG2D-mediated activation signals can override the inhibitory signals of NK inhibitory receptors; thus, NKG2D is recognized as the main activating NK cell receptor, and NKG2D ligand expression (NKG2DLs) is strictly controlled. Based on plasma analysis of 44 HNSCC patients and 12 healthy volunteers, HNSCC may secrete NKG2DLs to inhibit the activation of NKG2D receptors on the NK cell surface, impair NK cell anti-tumor activity, and invade tumors, leading to immune escape (46, 47). Exosomes in the blood of HNSCC patients also inhibit ADCC in NK cells by suppressing the expression level of NKG2D. Co-culture of exosomes isolated from venous blood samples of patients with acute myeloid leukemia (AML) or HNSCC and healthy volunteers with NK cells revealed that they inhibited NKG2D expression in CD3^−^CD56^+^ NK cells and significantly decreased the NKG2D^+^ NK cell frequency (48). HNSCC patient-derived exosomes also significantly enhance CD8^+^ T-cell apoptosis and suppress NK cell proliferation and NKG2D expression (49).

The use of immune checkpoint inhibitors may be one of the reasons why HNSCC evades immune surveillance. TIM-3, an inhibitory receptor on the surface of NK cells, suppresses NK cell function, resulting in cancer metastasis or recurrence in patients (50). KIRs are also regarded as key receptors that affect human NK cell development and function (51). Drugs targeting NK cell inhibitory receptors (e.g., KIRs) (e.g., lirilumab) have been the focus of preclinical studies, either independently or in association with other immune checkpoint inhibitors (4). KIR3DL2, a member of the killer cell immunoglobulin-like receptor family, blocks NK cell activation and function upon contact with HLA-A3 or HLA-A11 and also contributes to immune escape from HNSCC (52).

In addition, HNSCC secretes other cytokines and immune components in the tumor microenvironment (TME) to suppress or activate T cells, B cells, and dendritic cells, directly or indirectly regulating NK cells (53–56).

## 5 NK cell-based immunotherapy in HNSCC

Numerous studies have indicated that the number and function of NK cells are associated with prognosis in patients with HNSCC. The role of NK cell checkpoints in neoplasm immunotherapy has attracted considerable interest. Currently, most NK cell-based immunotherapies use NK cell function as an entry point to strengthen the ADCC effect of NK cells by activating or inhibiting NK cell surface receptors (**Table 2**).

**Table 2:** Summary of clinical trials with NK cells.

Clinical trial	N	Target	Treatment	Stage	Primary endpoint	Years		Ref
NCT02110082	66	CD37	Cetuximab/urelumab	Ib	Enhancement of cytotoxic and proliferative markers in NK cells	2014–2016		(57)
NCT01218048	40	Treg	Cetuximab	II	Monotherapies differentially activated NK cells Treg > 6% (poor prognosis)	2011–2017		(58)
NCT00226239	39	Treg	Docetaxel/cisplatin/cetuximab	II	Treg < 6% PFS extension	2005–2013		(58)
NCT01218048	40	PD-1 EGFR	Cetuximab operation Postoperative radiotherapy Cisplatin or carboplatin	II	NK cell proliferation Response increased after treatment	2011–2017		(59)
NCT02643550	143	PD-1	Monalizumab/cetuximab/anti-PD (L) 1	Ib/II	Evaluable efficacy (n = 26) RECIST partial reaction (n = 8) SD (n = 14), PD (n = 3), death (n = 1)	2015–2023		(60)
NCT02609386	105	CTLA4 PD-L1	IRX-2	IIb	Reduction in tumor volume (90%, n = 105) ADCC enhancement of NK	2015–2022		(61)
NCT01468896	23	IL-12	IL-12 Cetuximab	I/II	PFS > 100 days (n = 11) SD: 69%	2011–2015		(62)
NCT01334177	13	TLR	VTX-2337/cetuximab	Ib	ORR: 15% DCR: 54% NK cell number and activation were statistically significantly increased	2011–2014		(63)
NCT01836029	195	TLR	Motolimod/VTX-2337 Carboplatin/cisplatin 5-Fluorouracil	II	PFS (6.1 vs. 5.9 months) OS (13.5 vs. 11.3 months)	2013–2016		(64)
NCT02325401	20	CXCL1 STAT1	Metformin/cisplatin	I	NKG2D expression was increased NK cell tumor infiltration was enhanced Promote NK cell recruitment	2015–2020		(65)
NCT02110082	66	CD37	Cetuximab/urelumab	Ib	Enhancement of cytotoxic and proliferative markers in NK cells	2014–2016	(57)	
NCT01218048	40	Treg	Cetuximab	II	Monotherapies differentially activated NK cells Treg > 6% (poor prognosis)	2011–2017	(58)	
NCT00226239	39	Treg	Docetaxel/cisplatin/cetuximab	II	Treg < 6% PFS extension	2005–2013	(58)	
NCT01218048	40	PD-1 EGFR	Cetuximab operation Postoperative radiotherapy Cisplatin or carboplatin	II	NK cell proliferation Response increased after treatment	2011–2017	(59)	
NCT02643550	143	PD-1	Monalizumab/Cetuximab/anti-PD (L) 1	Ib/II	Evaluable efficacy (n = 26) RECIST partial reaction (n = 8) SD (n = 14), PD (n = 3), death (n = 1)	2015–2023	(60)	
NCT02609386	105	CTLA4 PD-L1	IRX-2	IIb	Reduction in tumor volume (90%, n = 105) ADCC enhancement of NK	2015–2022	(61)	
NCT01468896	23	IL-12	IL-12 Cetuximab	I/II	PFS > 100 days (n = 11) SD: 69%	2011–2015	(62)	
NCT01334177	13	TLR	VTX-2337/cetuximab	Ib	ORR: 15% DCR: 54% NK cell number and activation were statistically significantly increased	2011–2014	(63)	
NCT01836029	195	TLR	Motolimod/VTX-2337 Carboplatin/cisplatin 5-Fluorouracil	II	PFS (6.1 vs. 5.9 months) OS (13.5 vs. 11.3 months)	2013–2016	(64)	
NCT02325401	20	CXCL1 STAT1	Metformin/cisplatin	I	NKG2D expression was increased NK cell tumor infiltration was enhanced Promote NK cell recruitment	2015–2020	(65)	

NK, natural killer; PFS, progression-free survival; EGFR, epidermal growth factor receptor; RECIST, Response Evaluation Criteria in Solid Tumors; SD, stable disease; PD, progressive disease; ADCC, antibody-dependent cell cytotoxicity; ORR, objective response rate; DCR, disease control rate.

### 5.1 Monoclonal antibody

#### 5.1.1 Cetuximab

**Figure 3:**
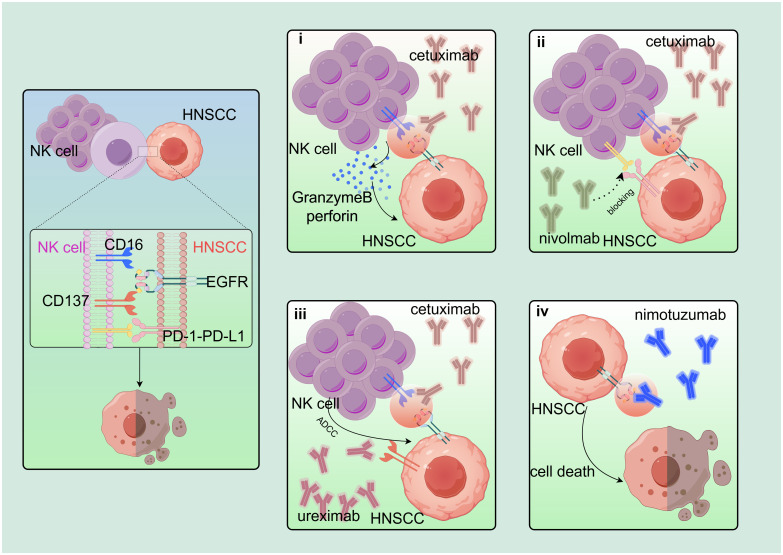
Based on the combination of cetuximab. EGFR-based combination therapy regimen: i) CD16 on NK cell surface is linked to EGFR on HNSCC surface via cetuximab to induce NK cells to secrete perforin and granzyme B to kill target cells. ii) Nivolumab blocks PD-1–PD-L1 axis and synergistically increases cetuximab treatment effect. iii) Cetuximab also stimulates NK cells to express CD137, and urelumab (agonist of CD137) acts synergistically with cetuximab. iv) Nimotuzumab binds to EGFR and blocks EGFR downstream signaling. By Figdraw. EGFR, epidermal growth factor receptor; HNSCC, head and neck squamous cell carcinoma.

Cetuximab is an epidermal growth factor receptor (EGFR)-specific chimeric immunoglobulin G (IgG)1 isotype monoclonal antibody approved for the treatment of HNSCC (**Figure 3**). After co-culture with UM-22B HNSCC in charge of cetuximab, the NK cells activation was increased, and NK cells expressing different genotypes of FC-γRIIIa receptor (VV, VF, and FF) were differently activated (66). Furthermore, after co-culture of TU167 HNSCC with NK cells in charge of cetuximab, NK cells containing FcγRIIIa V allele had significantly enhanced ADCC ability compared to NK cells containing only the F allele. This V-allele advantage was also observed in the 012SCC cell line (67). In a 2008 randomized clinical trial (NCT00122460), 442 eligible patients with untreated HNSCC were randomly assigned to receive cetuximab treatment while receiving the same chemotherapy. Compared with chemotherapy alone, cetuximab improved progression-free survival (PFS) from 3.3 months to 5.6 months, while the response rate increased from 20% to 36% (p < 0.001) (68). In another single-arm, multicenter, phase II trial (jRCTs051200040), combination chemotherapy with paclitaxel and cetuximab was administered to 35 recurrent/metastatic (R/M)-HNSCC patients previously treated with platinum-based chemotherapy and PD-1 (programmed cell death protein 1) antibody. Outcome was evaluated in 33 of 35 patients, with objective response rate (ORR) of 69.6% (95% CI, 51.2% to 84.4%) and disease control rate (DCR) of 93.7% (95% CI, 79.7% to 99.2%) (69).

#### 5.1.2 Lirilumab

HLA-C missense mutations are frequently observed in patients who do not respond to cetuximab. Normal HLA-C binds to and inactivates KIRs, thereby activating NK cells. Lirilumab is targeted to activate NK cell KIR mAb, and in charge of lirilumab, NK cells were cultured in HNSCC JHU029 and 93 vu to improve the NK cell killing effect (70). Nivolumab and lirilumab in an open-label, single-arm, multicenter, phase II trial (NCT03341936). A pathological response to N + L was observed in 43% (12/28) of patients, a major response (tumor viability TV ≤ 10%) in 4/28 (14%), and a partial response (TV ≤ 50%) in 8/28 (29%). The median follow-up time was 22.8 months, and the 1-year disease-free survival (DFS) and overall survival (OS) were 55.2% (95% CI, 34.8 to 71.7) and 85.7% (95% CI, 66.3 to 94.4), respectively. Among patients with a pathological response, the 2-year DFS and OS rates were 64% and 80%, respectively. DFS was significantly improved in patients with a pathological response, and this regimen should be further validated (71).

#### 5.1.3 Urelumab

Cetuximab also activates NK cells to upregulate the costimulatory receptor CD137. Cetuximab-coated JHU-029 HNSCC can increase CD137 expression on NK cell surface, and NK cells expressing FcγRIIIa VV/VF showed the most significant expression. Urelumab, a CD137 agonist, not only improved the survival rate of cetuximab-activated NK cells but also promoted dendritic cell (DC) maturation. Combination immunotherapy with cetuximab and CD137 agonists has shown beneficial effects (57).

#### 5.1.4 Ipilimumab

Treg inhibited ADCC and/or NKG2D NK cells expression in a TGF-β1-dependent manner. In 22 patients treated with cetuximab, Tregs in the peripheral blood were increased by comparing peripheral venous blood from patients 7 days before and 1 month after treatment. JHU029 HNSCC cells were added to NK cells cultured with cetuximab in a 1:1 ratio with ipilimumab, which enhanced the ADCC of NK cells by targeting CTLA-4 to eliminate Tregs (58). Definitive radioimmunotherapy (RIT) with nivolumab and ipilimumab for locally advanced (LA) HNSCC was administered in a phase I trial involving 24 patients. The 3-year PFS was 74% (95% CI, 58% to 94%), and OS was 96% (95% CI, 88% to 100%). However, 21 (88%) patients reported treatment-related adverse events, and level 3 or higher was likely due to the small sample size. Based on the 3-year PFS and OS results, definitive RIT with nivolumab and ipilimumab had superior efficacy compared to standard chemoradiotherapy with high-dose cisplatin (72).

#### 5.1.5 Nivolumab

JHU029 HNSCC cells were co-incubated with the treated (mAb) and freshly purified NK cells for 4 h. From the collected supernatant, we found that cetuximab-activated NK cells by PD-1 tended to be inactivated by the overexpression of PD-L1 (programmed death ligand 1) in the HNSCC TME. The effect of cetuximab was further inhibited by the downregulation of cell surface CD16. NK cells overexpressing PD-1 were co-cultured with three cell lines, SCC90 (PD-1^−^), JHU029 (PD-1^low^), and 93VU (PD-1^high^). It was found that nivolumab acted by blocking the PD-1-PD-L1 axis and significantly increased cetuximab-mediated killing (59). Nivolumab in combination with ipilimumab, with a complementary mode of action, demonstrates OS benefits and durable responses in a wide range of solid tumors. CheckMate 651 (NCT02741570) found that the median OS of nivolumab plus ipilimumab was 13.9 months (95% CI, 12.1 to 15.8). However, the OS for the EXTREME (≤6 cycles of cetuximab plus cisplatin/carboplatin plus fluorouracil, followed by maintenance cetuximab) was 13.5 months (95% CI, 12.6 to 15.2). CheckMate 651 does not meet the OS requirements, but compared with EXTREME, CheckMate 651 showed good security. Patients with R/M-HNSCC require new treatments (73).

#### 5.1.6 Monalizumab

The combination of monalizumab, a humanized anti-NKG2A antibody, with a PD-1-PD-L1 axis blocker enhances NK cell activity against a variety of tumor cells and rescues CD8^+^ T-cell function, a novel mechanism of checkpoint inhibition. It also promotes NK cell ADCC and cetuximab antitumor activity (74). The human HNSCC cell lines SNU-1041, SNU-1066, and SNU-1076 were cultured with NK cells. IFN-γ secreted via NK cells increased the expression of HLA-E on tumor cells. The HLA-E expression levels in SNU-1041 and SNU-1066 cells were higher than those in SNU-1076 cells. A combination of mAbs and cetuximab is more effective (60). In a phase II trial (NCT02643550), investigators evaluated the safety and efficacy of the combination of monalizumab and cetuximab in previously treated patients with recurrent or metastatic HNSCC. The midterm test results showed an ORR of 31%. The most common adverse events were fatigue (17%), fever (13%), and headache (10%). However, cetuximab monotherapy has limited activity in relapsed and/or metastatic HNSCC treatment, with an ORR of 13% (74).

#### 5.1.7 Durvalumab

Durvalumab is a kind of immunoglobulin G1 kappa monoclonal antibody, as an antagonist of PD-L1 signal, often associated with other monoclonal antibodies for solid tumor treatment (75, 76). In a phase 2 study of cetuximab plus durvalumab (NCT00122460), NK cells were evaluated for the treatment of ADCC. Cetuximab increased NK cell cytotoxicity and had a synergistic effect when durvalumab was added to responders. The ORR was 39% (13/33), and the median response duration was 8.6 months (95% CI, 6.5 to 16.8). Median progression-free survival was 5.8 months (95% CI, 3.7 to 14.1), and overall survival was 9.6 months (95% CI, 4.8 to 16.3). Of note, patients who had received ICI (pembrolizumab or nivolumab) had better OS than those who had not (median OS 7.1 months; 95% CI, 2.8 to not reached (NR) vs. 13.9 months; 95% CI, 4.8 to NR) (77).

#### 5.1.8 Avelumab

Thus, avelumab, combined with targeted therapy, may be a promising treatment option for cancer. After binding to tumor cells, avelumab activates NK cell ADCC to kill tumor cells by interacting with CD16 receptor/FcγRIII on NK cells by antibody Fc region (78). In a phase 3 trial, avelumab–cetuximab had an expected hazard ratio (HR) of 0.64 compared to cisplatin–RT. The HR for avelumab–cetuximab versus cetuximab was 0.62 (79). In another trial of avelumab plus chemoradiotherapy (NCT02952586), the median follow-up for progression-free survival with avelumab was 14.6 months [interquartile range (IQR) 8.5 to 19.6], and that of the placebo team was 14.8 months (11.6–18.8), which did not achieve the desired results (80).

#### 5.1.9 Camrelizumab

In a single-center, prospective, randomized controlled trial, responders had significantly higher NK cell counts than non-responders when camrelizumab was co-administered with chemotherapy. CD56^dim^ NK cell values can also be used to predict efficacy (81). In a single-center, single-group, phase 2 trial (chictr.org registry, ChiCTR1900025303), the PD-1 inhibitor camrelizumab was added to neoadjuvant chemotherapy. Thirty patients completed neoadjuvant therapy, with an ORR of 96.7% (29/30); 27 patients underwent immediate surgery, with an R0 removal rate of 92.6% (25/27) (82). In another phase I trial (NCT04393506), 20 patients with locally advanced resectable oral squamous cell carcinoma (OSCC) who received camrelizumab combined with apatinib had an 18-month locoregional recurrence rate of 10.5% (95% CI, 0% to 24.3%) and a survival rate of 95% (95% CI, 85.4%-100.0%), and major pathologic response (MPR) rate was 40% (8/20). Camrelizumab combined with apatinib showed promising MPR compared to pembrolizumab monotherapy (4.3%–20.5%), nivolumab monotherapy (5.9%), or nivolumab plus ipilimumab (20%) (83).

### 5.2 Interleukin

In addition to monoclonal antibodies, numerous cytokines have been found to augment NK cell function. Some of these interleukin family factors have been proven to upregulate NK cell activity and prolong NK cell survival *in vivo*.

Preclinical studies have demonstrated the anti-tumor immune potential of IL-2. In the early 1980s, IL-2-treated human peripheral blood mononuclear cells increased the number of killer cell populations (LAK), which were mainly composed of NK and T cells (84). IL-2, a confirmed activated NK cell ligand, is directly involved in NK cell antitumor activity. IL-2, the main component of the biological agent IRX-2, strengthens NK cell function by increasing the NKp30 and NKp46 expression levels (61, 85). Notably, high doses of IL-2 are life-threatening.

Over the past decade, IL-15 has gradually replaced IL-2. IL-15 can be used in NK cell therapy to increase lysis of antibody-coated target cells. ALT-803 is a novel compound consisting of genetically modified IL-15, and the IL-15 receptor alpha protein (IL15R) partially fused to IgG1 Fc. ALT-803 increased the ADCC effect in NK cells more strongly than IL-15 alone and, in combination with cetuximab, activated the JAK/STAT and MAPK signaling pathways in NK cells, resulting in stronger activation of cetuximab-coated HNSCC cells against NK cells. This treatment significantly reduced the size of tumors in mice (86). IL-21 also plays an important role in the regulation of T, B, and NK cells. Low concentrations of IL-21 and IL-15 synergistically promote NK cell growth, whereas high concentrations of IL-21 significantly inhibit NK cell growth. IL-21 induces humoral and cellular immune therapies and antitumor immune responses and causes no serious adverse reactions (87).

IL-12 has a similar structure and effect as IL-15, which can enhance NK cell ADCC in HNSCC cell lines and the dissolution activity of NK cells against cetuximab-coated cells simultaneously (62). Combined treatment with IL-2 and cetuximab significantly increases NK cell ADCC activity (88). Furthermore, IL-27 is an IL-12 family cytokine that enhances the ADCC of NK cells *in vitro* and *in vivo* and enhances NK cell viability *in vitro* (89).

### 5.3 PD-L1 t-haNK

Recently, increasing attention has been paid to the efficacy of engineered NK-92 cell lines in HNSCC therapy. Before clinical use, the NK-92 cell line derived from lymphoma patients must be subjected to deadly radiation. Adoptive transfer of the irradiated NK-92 cell line has proven to be safe and has shown preliminary clinical benefits in patients with cancer (90). NK-92 cells and their derivatives are designated as NK cells. aNK is modified to express IL-2 and CD16 with high affinity for PD-L1 (PD-L1 t-haNK). These cells not only preserve natural NK receptor expression but also contain high levels of granzymes and perforin granules (91, 92). PD-L1 t-ha NK cells induce cell lysis *in vitro* in a variety of human cancer cell lines (such as breast cancer, lung cancer, colon cancer, ovarian cancer, stomach cancer, and meningioma) (91). Furthermore, PD-1/PD-L1 pathways are involved in the immune escape mechanism of tumor cells and are key targets for cancer immunotherapy (93). PD-L1 t-haNKs also reduce the frequency of endogenous high PD-L1-expressing leukocytes in patients with HNSCC, an important complementary mechanism for killing tumor cells (94). Most importantly, the cytolytic ability of NK cells decreases under hypoxic conditions in most solid tumors, whereas haNK cells retain their cytolytic ability under hypoxic conditions (95). In addition to its direct HNSCC-killing effect, PD-L1 t-haNKs *in vitro* can be mediated by PD-L1 chimeric antigen receptor (CAR) to escape T cell-killing tumor cells (96). *In vitro* co-culture of peripheral blood leukocytes from advanced HNSCC patients with PD-L1 t-haNK for 24 h significantly reduced the number of macrophages and CD14^+^/CD15^+^ bone marrow cell subsets with high PD-L1 expression. The results showed that PD-L1 t-haNK holds therapeutic promise for patients with HNSCC with high PD-L1 expression (94).

### 5.4 Others

Toll-like receptors (TLRs) are key to the immune response to the medium, and TLR agonists are of great significance for enhancing the effect of HNSCC treatment (63). TLR8 is the main sensor of microbial invasion. The TLR8 agonist VTX-2337 (motolimod) can effectively activate TLR8, enhance NK cell function by indirectly activating NK cells through the stimulation of monocytes or myeloid DCs (mDCs), and increase tumor-directed ADCC in conjunction with cetuximab. The combination of VTX-2337 and cetuximab increased the clinical response rate in epidermal growth factor receptor-positive patients (97). In 2019, Wu et al. analyzed CAL27, CAL33, and UMSCC47 HNSCC after treatment with metformin and found that metformin prevented the development of HNSCC (98). In 2022, McKenzie Crist et al. found that the treatment of HNSCC patients with metformin-activated anti-tumor immunity enhanced ADCC, NK cell counts, STAT1 pathway stimulation, and subsequent NKG2D receptor expression. Metformin also increases NK cell cytotoxicity by inhibiting CXCL1 (65). Vitamin D deficiency is prevalent in HNSCC patients, and NK cell infiltration within the tumor and/or stroma significantly increases after oral vitamin D administration. In the study of Florian Bochen et al., NK cells were isolated from the peripheral blood samples of 11 vitamin D-deficient HNSCC patients, of whom nine vitamin D responders showed improvement in NK cell activity compared with pre-treatment (99). Vitamin D acts on NK cells to exert anti-tumor effects, but a 2013 *in vitro* study showed that vitamin D also inhibits HNSCC cell proliferation (100). Myeloid-derived suppressor cells (MDSCs) inhibit NK cell function in HNSCC, and orally bioavailable SX-682 inhibits MDSC trafficking, thereby significantly eliminating tumor MDSC accumulation and enhancing tumor invasion, activation, and NK cell efficacy (101). WEE1 kinase inhibitors and cell cycle checkpoints enhance the sensitivity of HNSCC cells to ADCC weakening of tumor cell resistance to granzyme B-induced cell death (102).

## 6 Future perspectives

Car-NK follows the success of Car T-cell therapy (103). Recent clinical trials investigated the use of Car in different cancer treatments to determine its efficacy (104). Car-NK not only has the disadvantages of Car-T but also has the advantages of easy access, short production time, and inhibition of the immune escape of cancer cells (105). The role of allogeneic NK cell infusion in hematological tumors has also been validated (27). However, the expansion of NK cells *in vitro* faces many challenges and has limited infiltration into solid tumors (29). The short half-life of NK cells and the decrease in their cytotoxic activity with age are also reasons for the limitations of CAR-NK cells (106).

The solid tumor-associated antigen EphA2, a receptor tyrosine kinase, is overexpressed in many solid tumors and weakly expressed in normal human tissues (107). In an *in vitro* experiment, the UM-SCC9 HNSCC cell line with EphA2-CAR ML NK cells after training improved significantly compared with the control group EphA2-CAR ML NK cells of HNSCC lethality (108). Trophoblastic cell surface antigen 2 (TROP2) is a transmembrane glycoprotein. Trop2 overexpression is associated with disease progression and poor prognosis in various cancers. The experimenter designed a TROP2 specificity of CAR (T-CAR), NK-92 cells and PCI-13 HNSCC tumor cell lines after the training, activate the E7-NK-92 cells-double expression of TCR/T-CAR. T-CAR enhanced E7-TCR-mediated activation and cytotoxicity in TCR-engineered NK92-derived cell lines (109). In another trial, anti-HER1 (EGFR) CAR-NK-92 cells showed enhanced killing, apoptosis, and degranulation of HNSCC cells (110).

CD44v6, a subtype of CD44, is a potential target for HNSCC therapy (111). Anti-CD44v6 CAR-NK cells were prepared from peripheral blood-derived NK cells from healthy donors using a γ retroviral vector. Compared with the original generation of undecorated NK cells, CAR-NK cells for a variety of HNSCC cell lines (UT-SCC-14, UT-SCC-42B, SCC-25) increased by two to three times (112).

## 7 Conclusions

NK cells are special immune cells that respond swiftly to tumor cells. Tumors have a much harder time escaping immune surveillance by NK cells than T cells. However, the treatment of HNSCC with NK cells still faces many challenges. Interactions between immune cells in the TME affect the number of NK cells and ADCC. To enhance NK cell effects, NK cell expansion and infusion *in vitro* and antibody–drug coupling (ADC) have shown great promise and will be further investigated in future trials. Synergistic therapy between monoclonal antibodies also needs further trials to verify its effectiveness and safety. In addition, the combination of mAbs with other therapies may involve adjusting the dose of mAbs to reduce the probability of adverse events.

The CAR structures of CAR NK cells have undergone many iterations and have made good progress in the treatment of hematological tumors. Compared to CAR-T cells, CAR-NK cells are highly non-specific and do not cause graft-versus-host disease (GVHD). However, it lags behind the treatment of solid tumors, especially HNSCC. However, the number of donor cells was too small for treatment with CAR-NK cells. The safety and effectiveness of amplification also need to be further investigated in future studies.

Recently, NK cell immunotherapy has become a popular research topic. NK cell-based immunotherapy has important implications for surgical resection, preservation of maxillofacial function, tumor metastasis, and recurrence in patients with HNSCC.
